# Structural Analysis to Determine the Core of Hypoxia Response Network

**DOI:** 10.1371/journal.pone.0008600

**Published:** 2010-01-19

**Authors:** Monika Heiner, K. Sriram

**Affiliations:** 1 Department of Computer Science, Brandenburg University of Technology, Cottbus, Germany; 2 Projet Contraintes, INRIA Rocquencourt, Le Chesnay, France; Virginia Tech, United States of America

## Abstract

The advent of sophisticated molecular biology techniques allows to deduce the structure of complex biological networks. However, networks tend to be huge and impose computational challenges on traditional mathematical analysis due to their high dimension and lack of reliable kinetic data. To overcome this problem, complex biological networks are decomposed into modules that are assumed to capture essential aspects of the full network's dynamics. The question that begs for an answer is how to identify the core that is representative of a network's dynamics, its function and robustness. One of the powerful methods to probe into the structure of a network is Petri net analysis. Petri nets support network visualization and execution. They are also equipped with sound mathematical and formal reasoning based on which a network can be decomposed into modules. The structural analysis provides insight into the robustness and facilitates the identification of fragile nodes. The application of these techniques to a previously proposed hypoxia control network reveals three functional modules responsible for degrading the hypoxia-inducible factor (HIF). Interestingly, the structural analysis identifies superfluous network parts and suggests that the reversibility of the reactions are not important for the essential functionality. The core network is determined to be the union of the three reduced individual modules. The structural analysis results are confirmed by numerical integration of the differential equations induced by the individual modules as well as their composition. The structural analysis leads also to a coarse network structure highlighting the structural principles inherent in the three functional modules. Importantly, our analysis identifies the fragile node in this robust network without which the switch-like behavior is shown to be completely absent.

## Introduction

Biological networks tend to be huge and too complex to directly undergo mathematical analyses. However, networks are often also inherently modular. Thus, prior to mathematical analysis, they are decomposed into functional modules to better understand the dynamics [1]. Even after the decomposition of the network into modules, quantitative analysis may be a challenge due to lack of reliable kinetic data. Therefore, the problems that require immediate attention to understand the dynamics of a network are (a) to develop formal tools to decompose a network into modules and to determine the core network, (b) to understand the functional role of each of these modules in a network without subjecting to any quantitative analysis, and finally the challenging part is (c) to identify both the molecular pathways (modules) that contribute to robustness and the fragile molecular nodes which – when knocked off – paralyze the total function of a network. Importantly, all the above questions are related to the structure of a network. Thus, there is a need for adequate mathematical tools to acquire insights into the properties of a network without having to resort to quantitative analysis.

Petri nets are known to be a powerful tool to perform structural analysis of a network in general [2]–[4]. They can be used to probe any type of biological networks, be it a metabolic, signalling, or transcriptional network – or even a combination of them. Petri nets provide a flexible modeling language. Mass flow and biochemical or genetic regulation mechanisms can be equally represented at arbitrary abstraction level, ranging from molecular via cellular level [5]–[7] to multi-cellular level, describing, e.g., developmental processes in multi-cellular pattern formation [8], [9].

A Petri net framework may support a family of related Petri net models, sharing structure, but being specialized by their kinetic information [10]: qualitative (time-free) place/transition Petri nets as well as various types of quantitative (time-dependent) Petri nets such as time, stochastic, continuous, or hybrid Petri nets. Structural Petri net techniques infer properties of complex biological networks independent of any kinetic parameters. Thus, they can be applied in any case, even if no or little kinetic parameters are known. Many Petri net software comes with built-in features for analysis, animation and discrete (stochastic) as well as continuous (deterministic) simulation, which allow to investigate a model in various complementary ways [11]. For case studies demonstrating a unifying framework integrating qualitative, stochastic and continuous Petri nets and related analysis and simulation techniques see [10], [12], [13]. On that account, Petri nets have nowadays been widely used within the systems biology community; for review papers see [14], [15], for a short introduction see Section Methods.

In this paper we show how structural analysis techniques of the Petri net theory can be effectively employed to decompose a network into modules, to determine the core network, and to identify fragile parts of the network. We do so by using a well-studied biological example – the hypoxia response control. This network is well documented and thoroughly studied both from experimental and simulation point of view. We have choosen this network to compare with the results of Yu *et al.* [16], who used the same network to explain pathway switching in hypoxia by extreme pathway analysis [17].

Starting from the two earlier works [16], [18] we begin by exploring the network's subsystem responsible for HIF-1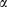 degradation using invariant analysis of Petri net theory. The invariant analysis reveals three modules that are capable of degrading HIF-1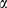. More importantly, it is found that the removal of those network parts not contributing to the I/O behavior and of the reversibility of the remaining reactions has no affect on the qualitative switch-like response of the network. Therefore, we have hypothesized that under certain conditions, reversible reactions are not necessary to achieve the required dynamics of the system. This in turn helps to reduce the number of dynamic variables and kinetic parameters in the system. Finally, we hypothesize that from invariant analysis it is possible to get a different perspective on the robustness of the network and the fragile element of the robust hypoxia response network. We will confirm these hypotheses by numerical simulations of the ordinary differential equations (ODEs) induced by reading the analysed qualitative Petri nets as continuous Petri nets with mass action kinetics. Importantly, we provide a different way of looking at robustness from the results of invariant analysis, which may be a promising and potential tool worth being further developed for a general theory of robustness.

The main contributions of our paper are:

- determination of the core module of the hypoxia response network, responsible for the switch-like behavior, which is shown to be behaviorally equivalent to the original network,
- deriving a coarse network structure of the core module, contributing to a better understanding of the network behavior,
- identification of the network redundancy, allowing for robustness, and of the network's fragile node, without which the switch-like behavior is shown to be completely absent,
- provision of a general approach for structural analyses, which can be equally applied to other networks, represented by any Petri net class (qualitative, time, stochastic, continuous, hybrid Petri nets). Remarkably, all steps are algorithmically defined.

## Results

In the following we give a short description of the hypoxia response network and related earlier modeling work. Afterwards we apply our structural analysis techniques to reduce the network structure. The reduced structure will be shown to be behaviorally equivalent to the original, full structure. The structural analysis techniques applied belong to the standard body of Petri net theory [2], [10], [19], recently supplemented by an algorithmically defined approach for network coarsening [20]. They are summarized in the Method section.

### Hypoxia Response Network

Oxygen is an essential and vital element for the survival of organisms. Lower oxygen content, termed hypoxia, arises under pathophysiological conditions like cancer due to low diffusion of oxygen to the tissue affected by tumor [21], while higher levels of oxygen, termed hyperoxia, leads to retinopathy of prematurity [22]. A well-studied molecular pathway activated under hypoxia condition is the Hypoxia Induced Factor (HIF) pathway. Key element of this network is the HIF transcription factor (TF) that exists in three forms, namely HIF-1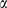, -2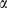, and -3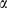. Under normal oxygenation, termed normoxia, HIF-1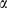 is constitutively degraded by 26S proteosomal system, whereas under hypoxia, HIF-1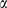 escapes proteosomal target and binds to Hypoxia Response Elements (HRE) which results in the activation of multiple target genes. In experiments it has been found that there is a critical concentration of oxygen below which HIF is present in large amounts, whereas above the critical concentration HIF is completely absent due to rapid degradation. Importantly, there is an inverse relationship between the level of oxygen content and HIF-1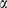 as observed in Hela [23] and Hep 3B cells [24].

**Figure 1:**
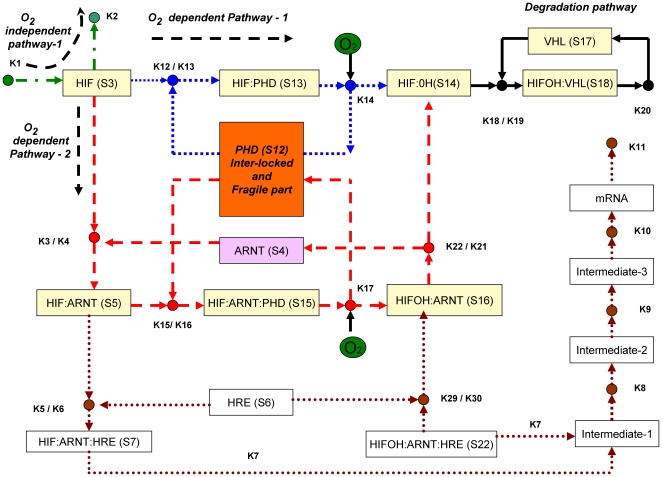
Schematic diagram of hypoxia response network. This scheme is based on references [16] and [18]. Three pathways, given in green (oxygen-independent pathway), blue and red (oxygen-dependent pathways) can degrade HIF transcription factor. The HRE activation pathway, shown in brown dotted lines, is – following the discussion in [16] – not considered in our analysis.

The schematic diagram in Figure 1 shows HIF-1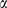 degradation by both oxygen-dependent and oxygen-independent mechanisms. Under the conditions of normoxia, the predominant pathway is binding of HIF-1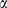 to prolyl hydroxylases (PHDs) which hydroxylates HIF-1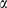 in the presence of oxygen followed by degradation by the von Hippel-Landau (VHL) protein. Degradation of HIF-1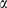 also takes place in an oxygen-independent manner, but with less efficiency. Under the conditions of hypoxia, HIF-1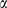 first saturates PHDs due to high binding affinity of PHD forming complex HIF-1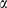-PHD, but cannot be hydroxylated due to low oxygen content and therefore cannot be degraded. The excess of HIF-1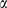 then binds to ARNT subunit and forms a complex HIF-1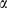-ARNT, which activates HREs. The complex also binds to PHD forming HIF-ARNT-PHD complex, and this also cannot be hydroxylated due to low oxygen content. The predominant form under hypoxia is HIF-ARNT that induces HREs. In summary, the switching behavior observed between normoxia and hypoxia is due to differential binding of HIF-1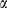 to PHD and ARNT subunit.

### Notations and Assumptions

We denote HIF-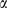 and HIF-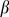 as HIF and ARNT, respectively. Even though we have not distinguished three different PHDs, it can be taken as PHD2 as *in vitro* studies have assigned the three PHD activities as PHD2
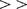
PHD3
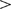
PHD1 [25] and *in vivo* studies have shown that PHD2 has a dominant role [26]. Further, PHD2 is mostly present in the cytoplasm even though it is capable of nucleocyctoplasmic shuttling. But in our work nuclear and cytosol compartments are not separately distinguished since (a) the core switching process, especially the reactions involving HIF-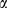, PHD and VHL, are known to take place in cytoplasm in many tissues [27], (b) even though the complex binding process of ARNT by HIF-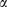-PHD complex takes place in the nucleus, the degradation by VHL is assumed to occur in the cytoplasm, and (c) no transcriptional regulation involving HRE is considered.

In summary, the transitional switching from hypoxia at low oxygen level to normoxia at high oxygen level is captured, and the intricate molecular mechanisms like transcriptional regulation during hypoxia or negative regulation during hyperoxia are not considered. Further, we have only considered the pathways that involve hydroxylation of proline residues and not asparagine residues. Therefore, new pathways like factor inhibiting HIF (FIH) that include the hydroxylation of aspargine residues or the occurrence of graded response mechanism involving reactions of Iron and prolyl hydroxylase are not considered [28], as a primary objective is to compare with the existing models of Yu et al. and Kohn et al. Detailed models including FIH can be found in the recently published article where the coarse grained molecular mechanisms of hypoxia “gene” regulation are considered [29].

Finally, as this is a first cautious step to use Petri nets as a tool to understand robustness and fragility through structural analysis, a rather simple network has been deliberately taken for illustration, even though the ultimate goal is to analyze complex networks. Therefore, the hypoxia network according to [16] is an illustrative example to study the viability of using Petri nets as tool for network analysis. Further work is needed to get insight about the role of other pathways in the hypoxia mechanism.

### Model Description

A theoretical model based on ordinary differential equations (ODEs) was first proposed by Kohn *et al.* [18] meant to capture the core subsystem responsible for the switch-like behavior of the hypoxia response network. Detailed analysis were later carried out by Yu *et al.* [16], who decomposed the network into several underlying pathways by extreme pathway analysis [17]. Analytical solutions for the decomposed network matched well with the solutions found by numerical integration (simulation). They concluded that pathway switching or pathway branching effect appears to be responsible for the sharp response to oxygen concentration.

**Figure 2:**
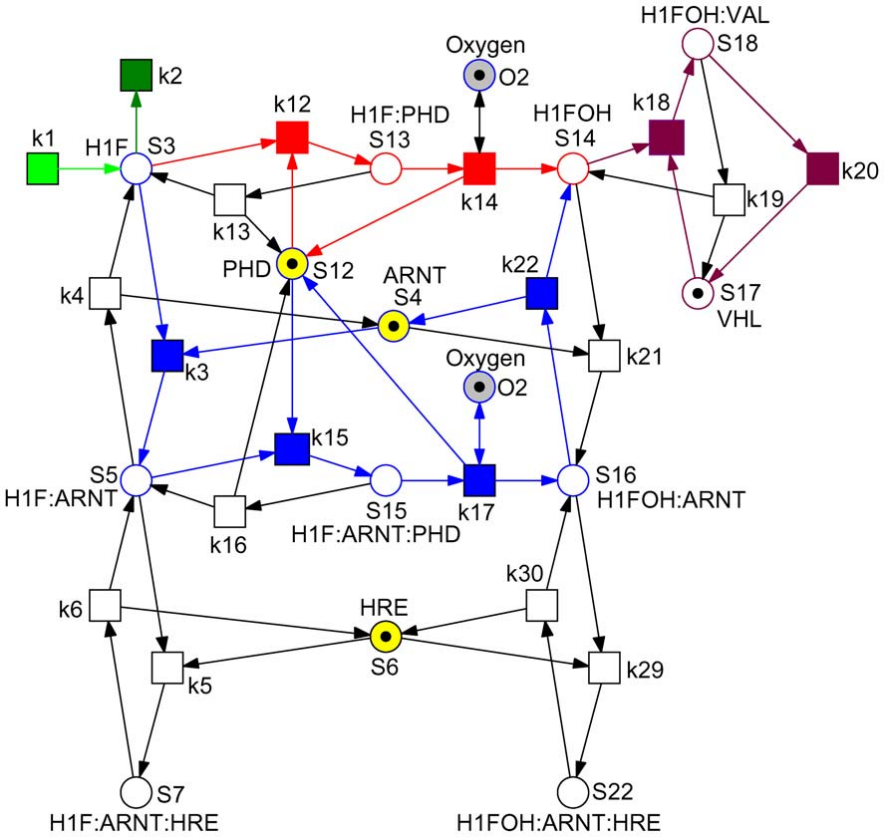
Petri net representation of the hypoxia response network. It defines equations (1–13), when read as continuous Petri net with mass action kinetics. The labels 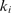 are taken as transition identifiers in the structural analysis of the qualitative Petri net, and as kinetic parameters (see Table 2) in the simulative analysis of the continuous Petri net. There are two logical places for 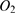, connected to the remaining net by read arcs. See Table 1 for the biological meaning of the other place identifiers (dynamic variables). Each color characterizes an ADT set, compare Table 5. Reduction candidates, as revealed by invariant analysis, are uncolored.

We take the ODEs model by Yu *et al.* and go one step further by demonstrating how to determine the core and the fragile node of the hypoxia response network by structural analysis. As the very first step, the network of Yu *et al.* [16] as given in Figure 1 is transformed into a Petri net, compare Figure 2. A closer look on Figure 1 reveals that the schematic diagram is already almost a bipartite graph (with a few exceptions), whereby rectangles (transitions) denote species, and small circles (places) reactions. So the transformation is straightforward and basically means swapping of places and transitions to adapt to the standard of biochemically interpreted Petri nets. Following the discussion in [16] we neglect the lower part of the schematic diagram.

To validate the transformation we generate the ODEs by reading the net in Figure 2 as a continuous Petri net [10], [30]. Applying mass action kinetics we get the equations (1–13), which exactly correspond to the equations given in [16]. The meaning of the dynamic variables is specified in Table 1. We keep all variable names as introduced in [16] to support comparison.

**Table 1:** Biological interpretation of places (dynamic variables) and initial concentrations.

Species	Place identifiers (Dynamic variables)	Initial concentrations (dimensionless)
**HIF**	**S** 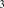	5 (0)
**ARNT**	**S** 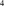	5
**HIF:ARNT**	**S** 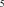	0
HRE	*S* 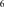	1
HIF:ARNT:HRE	*S* 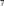	0
**PHD**	**S** 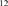	10
**HIF:PHD**	**S** 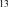	0
**HIF:OH**	**S** 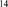	0
**HIF:ARNT:PHD**	**S** 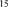	0
**HIF:OH-ARNT**	**S** 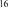	0
**VHL**	**S** 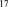	10
**HIFOH:VHL**	**S** 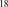	0
HIFOH:ARNT:HRE	*S* 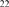	0

This table corresponds to Table 1 in [18]. The initial concentration given in brackets takes into consideration the results of P-invariant analysis, see Table 3. The continuous analysis yields the same steady state results for both initial concentrations. The variables of our core network – as derived by structural analysis – are given in bold.

Please note, there is no equation for 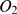. It only appears within the ODEs as side condition; so its concentration never changes for a given set of initial concentrations. The places 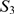 (HIF) and 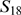 (HIFOH:VHL) model the input and output species.

We begin our analysis with the complete original network, consisting of 14 molecular species and 19 reactions and defining equations (1–13). The numerical simulations are carried out using the initial concentrations specified in Table 1. All three parameter sets as given in [16] yield the same qualitative results (see also Experiment 1). Our simulation results mirror exactly the results given in [16], which concludes the validation of the model transformation.

For further use we pick randomly the parameter set 1 from Kohn *et al.* [18], which is given in Table 2. The choice of parameter set does not have an influence on the reasoning, which we persuade in this paper.
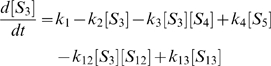
(1)

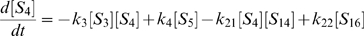
(2)

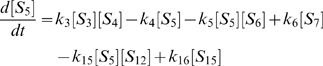
(3)

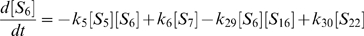
(4)

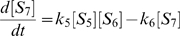
(5)

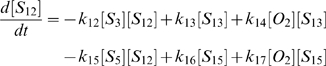
(6)

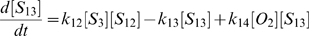
(7)

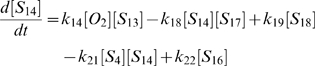
(8)

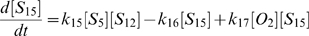
(9)

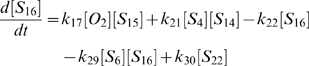
(10)

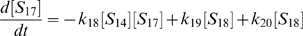
(11)

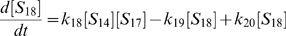
(12)

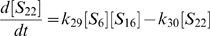
(13)


**Table 2:** Kinetic parameters.

Kinetic parameters	Values (dimensionless)
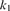	0.2321
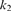	0.0017
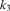 , 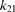	0.0121
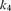 , 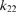	0.6163
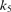 , 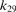	0.1693
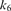 , 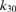	0.0566
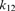 , 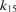	0.8326
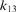 , 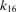	0.0196
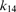 , 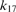	0.0361
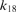	0.5722
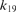	0.2667
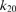	0.4591

Kinetic parameters as used in the simulation. This set corresponds to parameter set 1 in [18].

### Structural Analysis to Understand and Reduce Hypoxia Network

We start off with a biological interpretation of the network's P- and T-invariants as a complementary network validation step [31], before determining the core network.

#### Biological interpretation of P-invariants

It is good practice to validate a given network for expected properties before raising questions with unknown outcome. P-invariants belong to these established consistency checks. A P-invariant defines a mass-conserving subnetwork, i.e., a network in which the total mass is constant and therefore bounded. Each minimal P-invariant should enjoy a biological meaning, and – vice versa – there should not be an expected conservation law without corresponding P-invariant. See the Method section for more details, e.g. how to compute P-invariants.

The Petri net given in Figure 2 is not covered by P-invariants. However, it contains five P-invariants, which are listed in Table 3. Each of these P-invariants corresponds to a conservation law enjoying biologically sound interpretation.

**Table 3:** P-invariants.

index	P-invariant name	Places
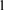	Oxygen	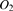
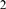	PHD 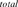	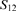 , 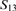 , 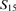
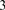	ARNT 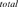	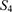 , 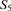 , 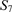 , 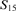 , 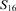 , 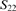
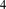	VHL 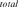	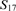 , 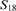
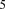	HRE 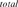	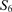 , 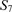 , 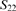

P-invariants (in set notation) of the Petri net given in Figure 2, each defining a mass conservation law. The places given first correspond to the non-complexed molecular species; so they get a non-zero initial concentration, compare Table 1.

The P-invariants in the hypoxia regulatory network arise due to the complexation of HIF with four important protein regulators: PHD (S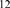), ARNT (S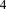), HRE (S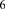), and the degradative protein VHL (S17), each of which results in a conservation relationship. Oxygen which plays a crucial role for our network under investigation is another obvious P-invariant on its own. Each P-invariant plays a vital role in the network and is therefore closely examined below. The order in which the P-invariants are examined is in accordance with the importance they play in the hypoxia regulatory network. The P-invariants found in the network can be broadly classified according to their role in normoxia and hypoxia. The following P-invariants (1–4) play a role in normoxia (where oxygen is involved), while the last one, (5), plays a role in hypoxia (oxygen-independent).

1. Oxygen (shown in green in Figure 1) is an important independent P-invariant in regulating HIF. The whole network dynamics depends on its critical concentration that *switches* the system from hypoxia to normoxia and vice versa. Therefore, maintaining critical concentration of oxygen is important for normoxia as shown in the numerical simulations of our computational experiments.
2. PHD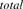 = PHD (S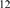) + HIF:PHD (S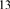) + HIF:PHD:ARNT (S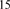)
   Oxygen cannot *per se* directly interact with HIF for degradation, but takes the help of other auxiliary proteins like PHD, ARNT and VHL. Among these three, PHD is important in complexing with HIF under the conditions of normoxia. In the absence of PHD, HIF persists, while in its presence, HIF is degraded by oxygen due to complex formation with PHD (S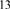). The higher the concentration of PHD, the efficient is the degradation of HIF, and as a consequence, critical oxygen concentration advances with the increase of PHD. This is due to high binding affinity of PHD to HIF that saturates HIF faster than ARNT. Even when ARNT (S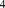) forms complex with HIF (S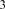), it is not degradable by oxygen unless the complex HIF:ARNT (S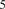) further complexes with PHD to give rise to HIF:ARNT:PHD (S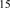) for the initiation of degradation by oxygen. Thus, PHD and its complexes that form a P-invariant are absolutely important for the network to maintain normoxia condition. PHD will indeed turn out to be the crucial node; knocking it off causes the whole hypoxia network to collapse.
3. ARNT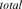 = ARNT (S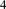) + HIF:ARNT (S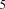) + HIF:ARNT:HRE (S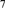) + HIF:ARNT:PHD (S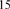) + HIFOH:ARNT (S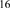) + HIFOH:ARNT:HRE (S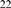)
   ARNT is another protein that has less binding affinity than that of PHD, opening a further pathway through which HIF is degraded. In the presence of PHD, the role of ARNT is very limited. Varying ARNT to higher concentration in the presence of PHD should not significantly change the critical oxygen concentration at which the system switches from hypoxia to normoxia. On the other hand, under absence of PHD, ARNT may play a significant role in degradation of HIF, but less efficiently in comparison to PHD.
4. VHL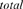 = VHL (S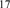) + HIFOH:VHL (S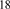)
   VHL is the protein that ultimately degrades hydroxylated HIF, which is the final step in the network for efficient normoxia.
5. HRE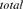 = HRE (S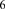) + HIF:ARNT:HRE (S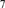) + HIFOH:ARNT:HRE (S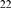)
   All the P-invariants (1–4) described so far are involved in normoxia for efficient degradation of HIF factors in the presence of oxygen. In the absence of oxygen, hypoxia response elements (HRE) play a vital role in invoking a further pathway. This constitutes the final P-invariant that plays a role in hypoxia.

Due to the conservation law, each P-invariant needs an initial concentration unequal to zero to bring the network to life, i.e. to involve all network parts into the game. Please note, this is a necessary, but not a sufficient condition.

The following places are not covered by a P-invariant, and consequently only bounded under suitable timing constraints: HIF (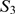), HIF:OH (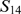). They are also not involved in a proper structural deadlock, i.e. in a set of places, which can never get marked again as soon as it got empty, see Method section for more explanations. So their initial concentration can be set to zero, see also capture of Table 1.

The mass conservation laws as determined by P-invariants can be exploited to automatically reduce the dimension of the ODEs generated for the continuous simulation experiments of a given continuous Petri net. This approach is common practice and not further discussed here.

The reasoning above will be confirmed by numerical simulations.

#### Biological interpretation of T-invariants

T-invariants are a further category which belong to the established consistency checks to support network validation before scrutinizing a network's dynamics. A T-invariant defines a state-conserving subnetwork, i.e., (a) a network whose transitions (reactions) bring the network back to a given state, if they all took place one after another (in the specified amount), or (b) keep the network in a given (steady) state, if all transitions permanently occur (in the specified relative frequency). The two transitions modeling the two directions of a reversible reaction always establish a T-invariant. Thus, it is called a trivial T-invariant.

Reading T-invariants as relative firing rates reproducing a given steady state allows us to identify superfluous network parts and those directions of reversible reactions not contributing to significant I/O behavior in the steady state. These are the transitions (reactions) not covered by non-trivial T-invariants.

A minimal T-invariant defines a minimal self-contained network behavior. These T-invariant-induced subnetworks are in the following called pathways. Likewise to P-invariants, each T-invariant should enjoy a biological meaning, and – vice versa – there should not be an expected pathway without corresponding T-invariant. Finally, each reaction should contribute to the network behavior. Therefore, the network should be covered by T-invariants. See the Method section for more details, e.g. how to compute T-invariants.

The Petri net given in Figure 2 is covered by T-invariants. First of all, there are the expected seven trivial T-invariants for the seven reversible reactions, which may be read as elementary futile association/dissociaten pathways: 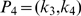, 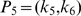, 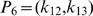, 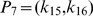, 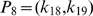, 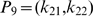, 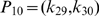.

Additionally, we get three non-trivial T-invariants, reflecting the I/O behavior, see Table 4. Please note, (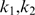) is not considered to be a trivial T-invariant, because it contributes to the I/O behavior. Each non-trivial T-invariant defines a pathway and can be thought of as a separate module contributing to the degradation of HIF. Among the three T-invariants shown in Table 4, the first T-invariant module is an oxygen-independent HIF degradation pathway (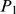), whereas two and three are oxygen-dependent pathways (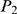 and 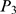).

**Table 4:** Non-trivial T-invariants.

T-invariant (Pathway)	Transitions (Reactions)
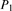	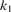 , 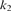
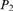	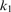 , 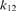 , 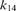 , 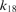 , 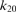
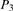	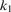 , 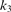 , 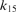 , 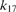 , 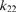 , 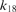 , 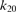

Non-trivial T-invariants (in set notation) of the Petri net given in Figure 2, each defining a pathway. Please note, (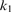, 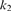) is not considered to be a trivial T-invariant.

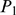
 – oxygen-independent HIF degradation pathway.
   This simple two-reaction pathway will turn out to be the least efficient pathway for HIF degradation.

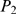
 – oxygen-dependent, PHD complexed HIF degradation pathway.
   This linear reaction pathway degrades HIF due to its affinity towards PHD which results in hydroxylation in the presence of oxygen. This pathway will be shown to be the most efficient one.

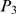
 – oxygen-dependent, predominantly ARNT complexed HIF degradation pathway.
   This pathway complexes HIF with both ARNT and PHD followed by hydroxylation in the presence of oxygen. Complexation of ARNT is important to invoke other pathways (cf. HRE) independent of PHD, in the absence of oxygen. This pathway will be located in terms of efficiency between 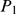 and 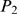.

In our network, these three pathways happen to be linear ones, i.e. the networks, induced by the transitions involved in a T-invariant, consist each of a non-branching path. Please note, this is generally not the case; for examples see [10].

**Figure 3:**
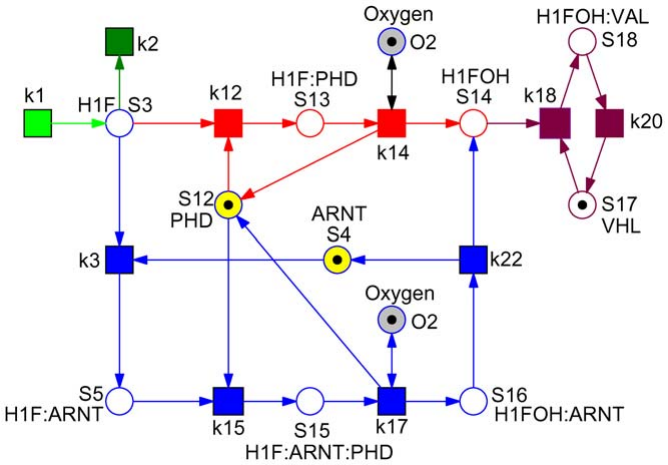
Hypoxia core network. All reduction candidates were automatically identified by structural analysis and got approved by the numerical simulation experiments of the corresponding ODEs. See Figure 4 for an hierarchical version of this Petri net.

The net is covered by T-invariants (CTI), however not covered by non-trivial T-invariants, (not strongly CTI). Transitions not involved in non-trivial T-invariants, and thus not contributing to the essential I/O behavior, are reduction candidates for steady-state-oriented reasoning. These are the transitions: 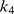, 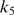, 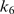, 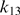, 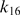, 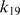, 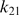, 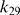, 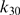, comprising the reversible reactions 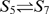, and 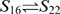, as well as the backward directions of all remaining reversible reactions. We get the core network shown in Figure 3, which requires 10 variables and 10 reactions, i.e. 3 variables and 9 reactions (kinetic parameters) less than the model used by Yu *et al.* in [16]. Based on our reasoning we *hypothesize* that it can still carry out its function efficiently, namely the switch-like transition from hypoxia to normoxia in the presence of oxygen. The model reduction will be validated by a number of numerical simulation experiments in the next section.

#### Biological interpretation of ADT sets

The final step in our structural analysis applies the concept of Abstract Dependent Transition (ADT) sets [20]. Two transitions depend on each other if they occur always together in the considered set of T-invariants. This obviously means that none of the two transitions can function without the other one; that's why they are called “to depend on each other”. Often we are interested in maximal ADT sets. However, for the application considered here we need connected ADT sets, i.e. ADT sets defining connected subnets (which is generally not the case, see [20] for examples). Thus, if required, we decompose maximal ADT sets into connected ADT sets.

Connected ADT sets provide a tool to hierarchically structure the identified T-invariants, which means at the same time to automatically coarsen a network. A connected ADT set can be abstracted by a macro transition (drawn as two centric squares), which will contain the replaced subnet on the next lower hierarchy level. We get an hierarchically drawn net, which is equivalent to the flat net. The crucial point is that the hierarchical description reveals the structuring principle inherent in the T-invariants, which may contribute to a better understanding of the network behavior. More importantly, it immediately allows us to identify redundant pathways and fragile nodes in the network structure, thus providing the grounds for a formal reasoning on a network's robustness.

**Figure 4:**
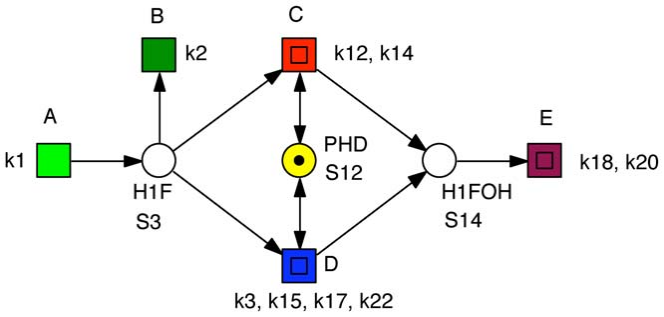
Coarse Petri net structure of the hypoxia core network. This is the top level of an hierarchical representation of the Petri net given in Figure 3. It reveals the structuring principle inherent in the minimal T-invariants. Each macro transition (drawn as two centric squares) stands for a connected subnet defined by a set of dependent transitions, i.e., transitions occurring together in all non-trivial T-invariants. A, B stand for 1-elementary sets, compare Table 4. The places shown in the coarse net structure are the interface places between the subnets. The coarse net structure clearly identifies the central role of PHD (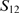). Its knock-down would switch off 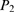 and 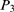. See also Figure 5.

Considering the three non-trivial T-invariants given in Table 4, we find six ADT sets, compare Table 5. Each ADT set induces a connected subnet; so, no further decomposition is required. We abstract each ADT set by a macro transition, and we obtain the coarse network structure as given in Figure 4.

**Table 5:** ADT sets.

ADT-set	Transitions
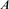	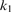
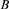	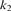
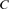	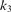 , 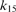 , 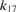 , 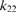
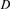	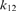 , 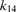
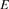	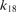 , 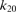
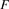	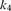 , 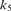 , 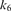 , 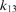 , 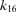 , 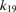 , 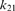 , 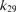 , 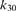

ADT sets as computed based on the minimal T-invariants, given in Table 4. Compare also Figure 3.

**Figure 5:**
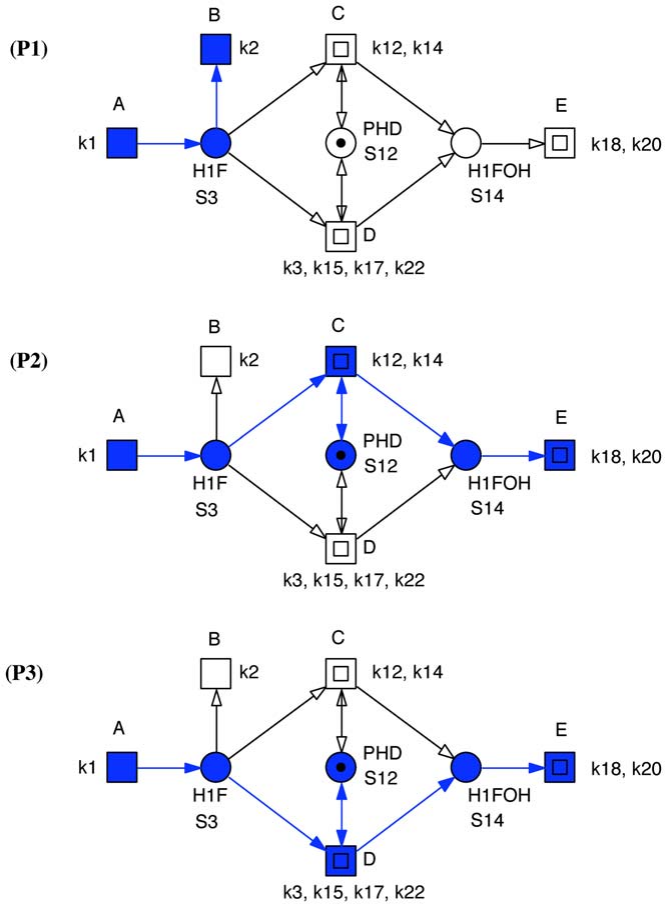
Pathways of the hypoxia core network. In this example, each macro transition path in the coarse net structure corresponds to a non-trivial T-invariant (pathway) of the flat network. There are three such sequences: 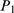 – direct degradation: (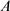, 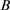), 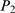 – degradation not requiring ARNT (S4): (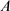, 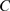, 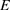), and 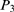 – degradation requiring ARNT (S4): (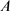, 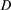, 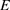).

Now, each macro transition stands for a connected subnet defined by a set of dependent transitions, i.e. transitions, occurring always together in all non-trivial T-invariants. Each elementary (loop-free) macro transition sequence in the coarse net structure corresponds to a non-trivial T-invariant of the flat network. There are three such sequences 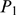 = (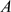, 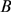), 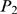 = (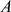, 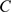, 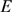), and 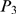 = (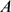, 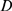, 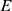), compare Figure 5. Please note, in our network, these three pathways happen to be linear ones, which is generally not the case. The places shown in the coarse net structure are the boundary places of the subnets, building the interface between the subnets. Thus, the coarse network highlights the structuring principle inherent in the non-trivial T-invariants (pathways).

Moreover, the coarse net structure elaborates clearly the redundancy among pathways 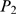 and 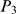 as well as the central role of PHD in the core hypoxia network, which both are not as obvious in Figure 3. The absence of PHD will result in an accumulation of HIF, because degradation by pathways 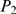 or 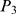 is precluded. As the concentration of PHD is increased, the efficiency of the HIF degradation in the presence of oxygen is expected also to increase, which needs to be checked by numerical simulation. Thus, PHD is the fragile node in the network, which – when knocked off – will result in a total loss of the robust switch-like behavior of the network.

In the next section we validate the conclusions from our structural analysis, particularly the hypothesis of the robustness of the identified core network, by numerical simulations.

### Validation of Model Reduction by Numerical Simulations

All ODEs subjected to numerical simulation in the following computational experiments are uniquely defined by reading the qualitative Petri nets discussed in the preceding section as continuous Petri nets with mass-action kinetics.

#### Reduction of original model to core model. Experiment 1

**Figure 6:**
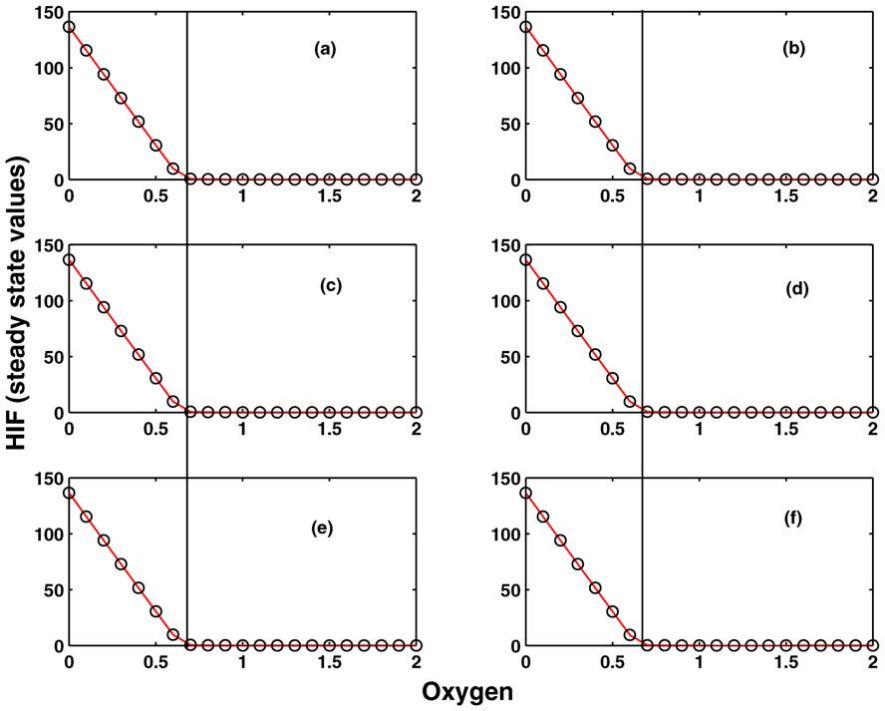
Experiment 1. Comparison of the dependency of the steady state value (SSV) of HIF (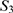) on the oxygen concentration by systematic silencing network parts as determined by T-invariant analysis by setting sequentially kinetic parameters to zero. (a) full model according to [16]; (b) kinetic parameters set to zero: 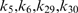; (c) 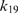
+(b); (d) 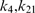
+(c); (e) 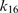
+(d); (f) 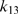
+(e). The experiment has been done for all three parameter sets as given in [18]. The dark vertical black line indicates the critical oxygen concentration (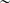
0.65) for which HIF is completely degraded. It separates hypoxia (left) from normoxia (right). The SSV and the critical oxygen concentration are the same in (a)–(f). Therefore, (f) is considered as the core module for further analysis.

We designed a number of experiments to validate the step-wise model reduction as suggested by the structural analysis and compare the results with the numerical simulation of the original model, which is shown in Figure 6(a). There are two aspects which need to be compared with the original model: (a) the Steady State Value (SSV) of HIF when oxygen is completely absent, and (b) the critical concentration of oxygen, which completely degrades HIF, i.e. the oxygen concentration at which the SSV of HIF goes to zero. The critical oxygen concentration is the concentration where the transition from hypoxia to normoxia or vice versa takes place.

In Figure 6, the steady state values of HIF concentration are plotted against oxygen to determine the critical oxygen concentration. In the original model, the SSV of HIF is ∼140 units when oxygen is absent, and the critical oxygen concentration is ∼0.65 at which all HIF is completely degraded.

We start off with systematic silencing network parts as determined by T-invariant analysis by setting the kinetic parameters one after the other to zero, compare Figure 6(b–f). This network is an I/O system with HIF as input and VHL as output. The oxygen regulates the pathway from HIF to VHL, and hypoxia or normoxia occurs depending on its concentration. In response to the concentration variation in HIF, the transcriptional cascade HRE becomes active, but is not directly involved in the core regulation of hypoxia or normoxia pathways. Therefore, silencing the HRE pathway has no direct implication on the degradation of HIF by oxygen and therefore the oxygen critical concentration where the transition from hypoxia to normoxia occurs should remain the same as in the full original model. As a result, the kinetic parameters 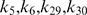 are set to zero, and as predicted by the structural analysis and confirmed by Figure 6(b), the critical oxygen concentration is the same as in the original model. This validates the assumption that HRE-related network part is not required for the core of the hypoxia response network.

After silencing the HRE network part, we looked at the remaining reversible reactions in the network, which may have any effect on the HIF degradation and the critical oxygen concentration. We silenced systematically the reversible reaction rate constants 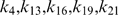 and it was confirmed that there is no impact on SSV's of HIF and the critical oxygen concentration, see Figure 6(c–f). This validated the reduction of the full model to the core model shown in Figure 3, which we obtained by the Petri net analysis and also support our hypothesis that the robustness of the whole network can be captured by studying the core network model, where the switch-like function is retained even after silencing 3 variables and 9 reactions in the network.

#### Numerical analysis of individual pathways

We continued our T-invariant-based validation with ordering the modules in terms of their importance for the robust switching.

#### Experiment 2

**Figure 7:**
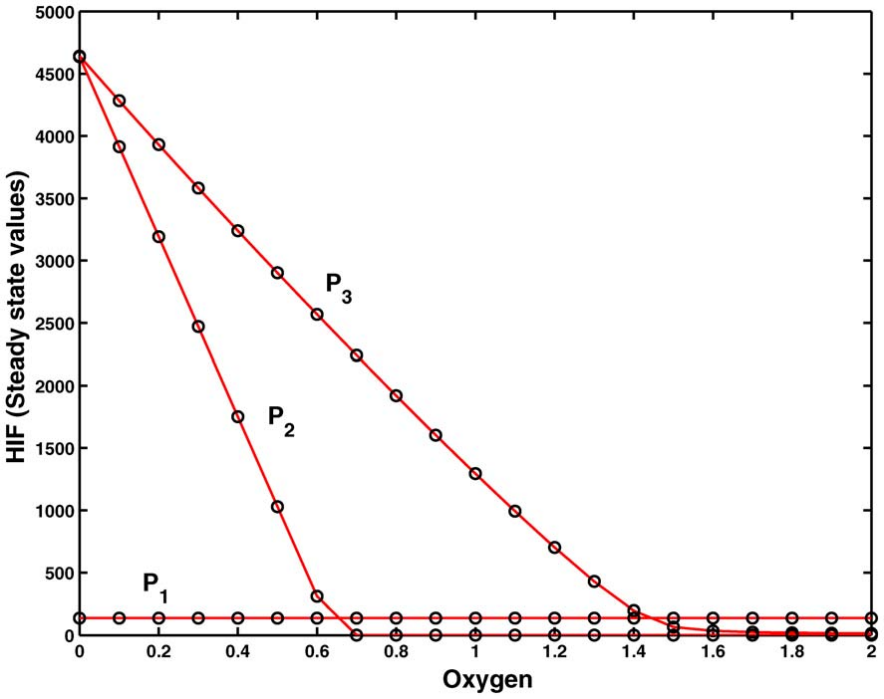
Experiment 2. Contribution of the individual pathways as induced by the T-invariants 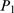, 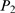 and 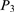, see Table 4, determined by numerical integration of the ODEs defined by each pathway. The efficiency of degradation of HIF by oxygen can be ordered as 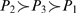. 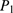 is the oxygen-independent pathway. 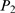 degrades more efficiently due to stronger binding of PHD to HIF than ARNT.

The T-invariant analysis identified three pathways, 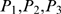, to be involved in the HIF degradation. 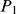 is an oxygen-independent HIF degradation mechanism, whereas the other two are oxygen-dependent mechanisms, where complexation – hydroxylation by oxygen – followed by degradation by VHL occurs in a step-wise fashion. Numerical simulation revealed that even though 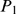 pathway degrades HIF, it cannot completely degrade on its own, and a constant amount of HIF is always present if oxygen is absent, see Figure 7.

On the other hand, pathway 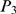 requires more oxygen to completely degrade HIF than pathway 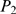. In both oxygen-dependent pathways PHD is involved in complex formation. But in 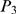, there is a competition with ARNT that prevents PHD to directly complex with HIF. Here, PHD plays only a supportive role for ARNT for hydroxylating the complex HIF:ARNT:PHD, and as a consequence it requires approximately twice the amount of oxygen than the 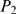 pathway for an efficient HIF degradation.

#### Experiment 3

**Figure 8:**
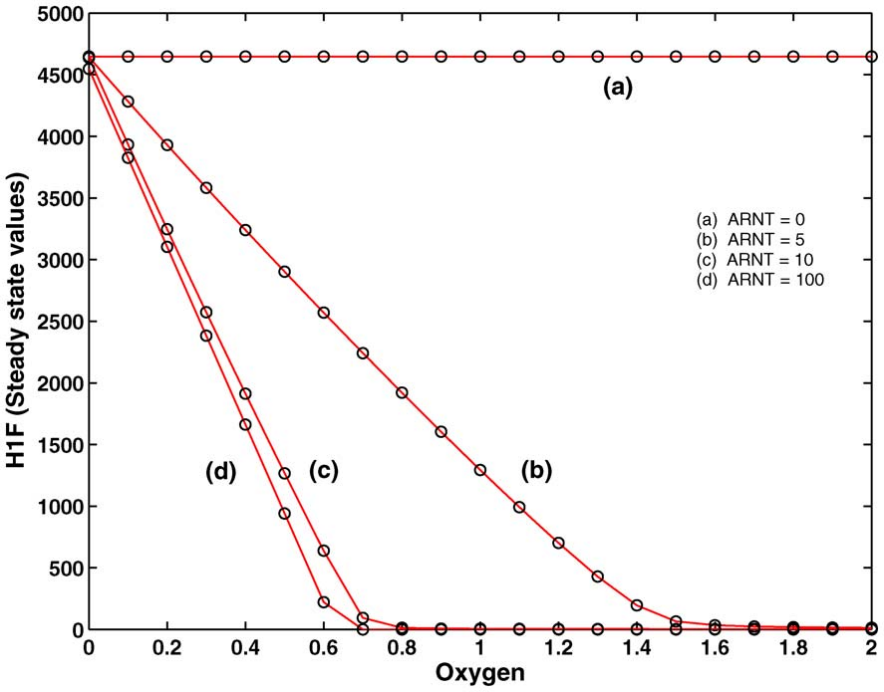
Experiment 3. Pathway 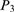 is considered separately while varying ARNT initial concentration (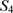). Degradation of HIF through pathway P3 is feasible for low (5) and medium (10) initial concentration of ARNT. For extremely high concentration (100), saturation of HIF by ARNT takes place and thereby the critical oxygen concentration (complete degradation of HIF) does not change.

We also explored the situation when ARNT binds much faster than PHD. This is captured by considering the pathway 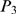 separately, such that the fast complexation between HIF:PHD is prevented. In this case, PHD can only bind to the complex HIF:ARNT which supports the hydroxylation reaction for further reacting with VHL for HIF degradation. In the absence of ARNT, HIF is not degraded and present in very high concentration. At a very low ARNT concentration, there is no sufficient HIF:ARNT complex, and therefore the degradation is less efficient. But at a very high ARNT concentration, the degradation is efficient, see Figure 8. This confirms that the 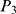 pathway which involves ARNT is less efficient than 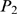. Therefore, it suggests that ARNT plays a different role, such as to invoke HRE rather than its support of oxygen-dependent HIF degradation.

In summary, the numerical analysis shows that the efficiency of the individual modules involved in the HIF degradation can be specified as 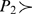 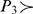 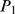. It also guides us to PHD as the fragile node, which seems to be the crucial point for the transition between hypoxia and normoxia. Therefore, we probed the role of PHD in the hypoxia network by numerical simulations in the next section.

#### Importance of PHD initial concentrations: the fragile node in the network. Experiment 4

The core model that was further hierarchically structured to a coarse network according to the structuring principle inherent in the non-trivial T-invariants, see Figure 4, revealed PHD as bridge between two different I/O pathways; i.e., PHD is a bridge between the two ADT sequences (A,C,E) and (A,D,E), both being oxygen-dependent.

**Figure 9:**
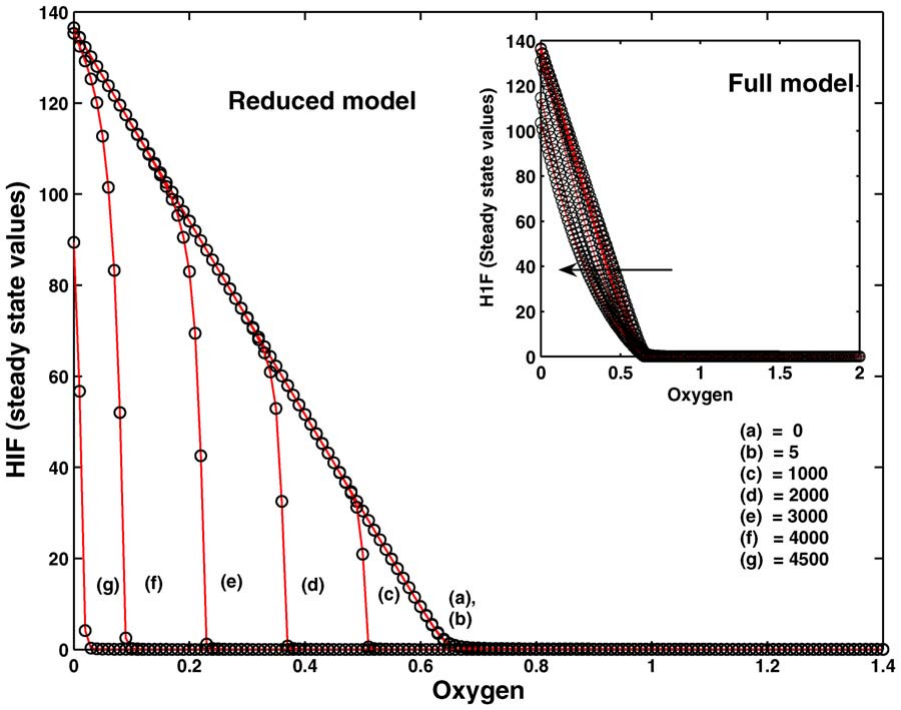
Experiment 4. Varying the initial concentration ARNT (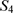) in the reduced model to a wide range (0, 5, 1000 … 4500). SSV and critical oxygen concentration are not affected for a very low value of ARNT initial concentration. Extreme concentration of ARNT (1000, 2000 …) which is 100 or more times than that of PHD initial concentration (10) changes both the SSV and the critical oxygen concentration to a lower value and as a result the curves are shifted to the left. We also give the simulation of the full model for varying concentration of ARNT. The concentration change is indicated by the direction of the arrow. The qualitative behavior is the same as for the reduced core model. Even for very high ARNT concentration the critical oxygen concentration is retained, but the HIF steady state values are lowered.

In the original model ARNT has a lower affinity to HIF than PHD. Therefore, the efficient degradation of HIF is still possible even in the absence of ARNT, as all HIF will be strongly bound by PHD to form HIF:PHD complex at a faster rate than HIF:ARNT complex. To validate this, we simulated our core model for various initial concentrations of ARNT. Figure 9 shows that the SSV's and critical oxygen concentration values remained the same as in the original model for very low initial concentrations of ARNT. Importantly, in the absence of ARNT concentration, the SSV and critical oxygen concentration remained also the same. At very high concentrations, the SSV and critical oxygen concentration moved to the left because competition of PHD is overruled by the presence of excess ARNT concentration, and thus both SSV and critical oxygen concentration fall at much lower values.

These simulation results suggest that the ARNT-dependent pathway 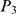 is not required for HIF degradation, and it only supports the degradation of HIF-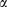 along with PHD. However, this redundancy contributes to the network's robustness.

#### Experiment 5

**Figure 10:**
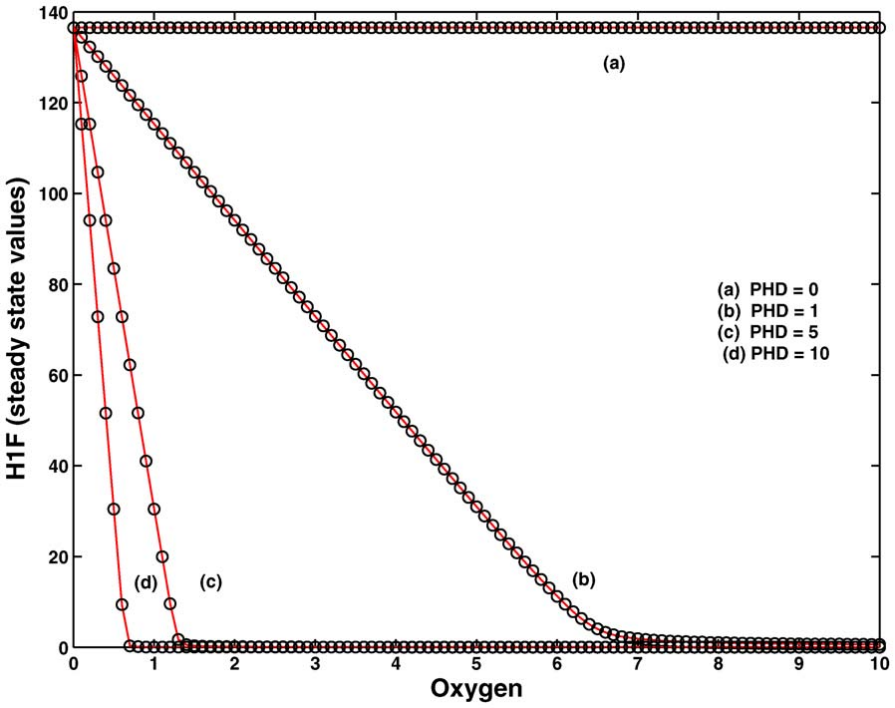
Experiment 5. Demonstration of fragile node PHD (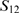) in the core module of the network. Increase in PHD concentration sHIFts the critical oxygen concentration to a lower value delineating the importance of binding affinity of PHD-HIF complex for degradation. The loss of PHD knocks off the oxygen-dependent degradation pathways 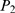, 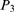 and results in inefficient degradation by the oxygen-independent pathway 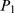 only, suggesting that PHD is the fragile node in the core module.

We also validated numerically that PHD is crucial for the degradation of HIF, and both SSV and critical oxygen concentration are extremely sensitive to the initial concentration of PHD, see Figure 10.

In the absence of PHD, HIF is not degraded, indicating that the HIF:PHD complex formation is crucial for the degradation of HIF. As the initial concentration of PHD is increased, the complexation increased proportionally, and the degradation became more efficient. This also points to the fact that PHD is the fragile node in the network, which, when knocked off or mutated, causes the whole system to collapse. In other words, the robustness of the whole network depends on PHD that ensures proper functioning of the network.

In an elegant experiment carried out by Pouyss*è*gur's group [26], it has been shown that silencing PHD2 with siRNAs is sufficient to stabilize and activate HIF-1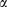 in normoxia, while PHD1 and PHD3 have no effect on the stability of HIF-1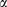. This suggest that PHD2 is the critical oxygen sensor maintaining low HIF-1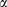 during normoxia and acting as fragile node in the network as captured by our analysis.

### Tools

The Petri nets have been drawn with Snoopy [32], available at http://www-dssz.informatik.tu-cottbus.de, which supports – among others – the design, animation and simulation of hierarchical qualitative and continuous Petri nets as used in this paper. The structural analyses have been done using Charlie [33], a tool to analyze qualitative Petri nets. Node sets computed by Charlie, such as P/T-invariants, or ADT sets, can be visualized by automatic coloring in Snoopy, which supports their manual evaluation. The automatic coarsening is subject of a running student's project.

All numerical simulations were carried out in MATLAB using the built-in variable step size ODE15s integrator with high absolute (10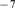) and relative (10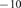) error tolerances. The simulations were done for a long simulation time period to avoid transients so that the system settles down to the steady state.

All our results are reproducible by help of the supplementary material, which we provide on our website.

## Discussion

### Structural Analysis and Reduction

In our work, we started from the ODEs model introduced by Yu *et al.* in [16] as a reduced version of the model presented by Kohn *et al.* in [18]. Our structural analysis approach identifies additional network parts not contributing to the significant I/O behavior in the steady state, and thus suggesting a further reduction of the core network. These are the transitions (reactions) not covered by non-trivial T-invariants, and any places (molecular species) getting isolated by deleting the reduction candidates. We identified 9 reactions and 3 molecular species as not being essential for the core model of the hypoxia response network, which reduces significantly the number of variables and parameters in the numerical analysis of the ODEs model of the network.

We confirmed the model reduction as suggested by structural analysis by a number of numerical simulation experiments where the corresponding reactions are knocked off one after the other. We demonstrated by further numerical simulation experiments that our reduced model exhibits exactly the same significant behavior as the model investigated in [16]. The core model identified by our structural analysis consists of 10 dynamic variables and 10 reactions. The model considered to be the core model in [16] consists of 13 dynamic variables and 19 reactions.

Furthermore, we derive a coarse network structure, highlighting the structural principle inherent in the given set of non-trivial T-invariants, and therefore in the pathways induced by them. This coarse network structure contributes to a better understanding of the network behavior, and – even more importantly – allows the clear identification of the fragile node in the network.

### Role of P-Invariants

We also did simulations with zero and non-zero initial concentration of the P-invariants (details not reported in this paper). If the initial concentrations of the invariants ARNT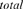, HRE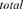, and VHL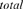 (compare Table 3) are set to zero, i.e., 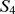 = 0, 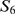 = 0, 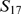 = 0 (compare Table 1), HIF can still be completely degraded at the critical oxygen concentration of 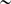
0.65, and switch-like behavior still persists.

Setting a P-invariant to zero is just another way to switch off certain parts of a network. Setting ARNT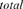 to zero is equivalent to switching off pathway 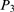. Setting HRE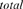 to zero is equivalent to switching off the subnetwork identified by our structural analysis as not contributing to the I/O behavior, as we did in our reported computational Experiment 1 by setting the kinetic parameters 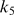, 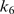, 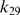, 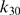 to zero. Setting VHL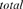 to zero will cause an accumulation of mass on 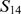, i.e. the actual final degradation step by 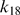 will be precluded.

However, setting Oxygen and PHD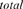 to zero would switch off the network parts crucial for the network behavior: 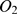, and the fragile node PHD. To activate the given network, it is sufficient to set the first two P-invariants (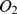, and, for example, 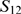) to non-zero initial concentrations. Otherwise, the network is completely paralyzed, i.e. switch-like behavior is absent, even when the other three invariants (ARNT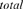, VHL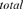, and HRE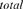) have non-zero initial concentrations. In summary, playing with the P-invariants' initial concentration is just another perspective to reason about the core network.

The ODEs defined by our core model could be further reduced for the continuous simulation experiments by exploiting the mass conservation laws as determined by three P-invariants (P-invariant 1 corresponds to a constant in the ODEs, P-invariant 5 is not part of the core network). In fact, the detailed analytical investigation undertaken in [16] have extensively exploited the conservation laws to derive a simple equation to determine analytically the critical oxygen concentration that exhibits switch-like behavior.

### Comparison with EPA

The hypoxia model is analyzed in [16] using the notion of extreme pathways [17]. Our structural analysis is based on T-invariants – one of the Petri nets' standard notions from the very beginning [19]. T-invariants and extreme pathways are closely related concepts, and both notions coincide for networks without reversible reactions. Sometimes it is a matter of choice, whether two reactions are considered as the two opposite directions of one reversible reaction, or just two closely related reactions, such as association and dissociation. However, T-invariants do never neglect trivial T-invariants which reflect exactly such local reproduction effects. Due to the analogy of both notions, our structuring approach, which we explained in this paper using the Petri net terminology, can be equally applied to the notion of extreme pathways and other related concepts.

### Robustness Vs. Fragility

Robustness and fragility are two sides of the same network. Robustness is defined as the ability of the system to maintain its function against internal and external perturbations [34], [35]. Therefore, to explore robustness it is paramount to identify the system, its function and the perturbations [36]. For the hypoxia network it is important to maintain its function, i.e. the switch-like behavior, against internal and external perturbations [37].

Redundancy is the hallmark of biological networks where the very same function is carried out by different pathways, which provides robustness against perturbations like mutation. If a mutation blocks an arterial pathway of a network, the network may still function due to the presence of an alternative pathway, and this is termed redundancy. Therefore, redundancy of a biological network is defined as the ability of the network to carry out the same function through different pathways to cope up with failures [38].

Identifying all the pathways that carry out the same function in a complex and large network is generally hard to achieve by visual inspection only. Thus we applied structural analysis, specifically T-invariants and the concept of ADT sets, to the hypoxia network to determine the core network and its pathways of HIF degradation, and to derive an hierarchically structured representation of the reduced network yielding the coarse network structure. The structure of the coarse network clearly elaborates redundant pathways by alternative paths between input and output species. Thus, the three different linear pathways involved in the degradation of the protein HIF-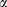 have been identified, out of which two are oxygen-dependent pathways which are responsible for the switch-like robust function. Another information that is teased out from the coarse network is that PHD is the shared node for the two oxygen-dependent pathways, which – when silenced – results in a complete loss of function of both oxygen-dependent pathways in the core network indicating that it is indeed the fragile node in the network.

We define a fragile node as a node which – when silenced – will paralyze all the identified redundant pathways, such that the expected switch-like behavior is completely lost. There may be some other nodes which when knocked off can paralyze parts of the network, but the switch-like robust behavior persists. Consequently, these nodes cannot be considered as fragile nodes. To illustrate this, ARNT is not a fragile node because, when it is silenced in the core network, a robust-switch like response is still preserved, because of the presence of the ARNT-independent pathway.

We have also shown for the first time by our case study that Petri nets may be used as a qualitative tool to determine the robustness of the structure of the network by identifying redundancy within the network that can be validated by numerical analysis. The modules that contribute to the I/O behavior can be easily extracted in terms of T-invariants, and as a consequence insight can be gained about the contribution of each module to the robustness of the network.

In the future, we would like to formalize the notion of robustness in Petri nets terms, complemented by further qualitative analysis techniques.

### General Procedure

The following steps summarize the general methodology which we have demonstrated by help of the hypoxia network as running example.

- Compute the minimal P-invariants and check their biological plausibility. Check whether each P-invariant gets a non-zero initial marking (concentration).

 Check whether the network is covered by P-invariants. Places not involved in any P-invariant may be structurally unbounded. They can be initially set to zero if they are not contained in a structural deadlock. The actual boundedness degree of such places, e.g. in a steady state, depends on the given timing constraints.

- Compute the minimal T-invariants and check their biological plausibility.

 Check whether the network is covered by T-invariants. Transitions not involved in any T-invariant may occasionally occur, but usually stand for particular modeling assumptions.

- If the model aims at the description of steady state behavior, check the network also for trivial T-invariants, and whether the net is covered by non-trivial T-invariants. Transitions not contained in non-trivial T-invariants are reduction candidates, as well as any places getting isolated by deleting the reduction candidates. Confirm by computational experiments the validity of the reductions.
- Compute the maximal ADT sets and check their biological plausibility.

 Compute the connected ADT sets and the interface places between them. Hierarchically re-structure the network by hiding each subnet induced by a connected ADT set in a macro transition.

- Evaluate the coarse net structure. Identify pathways redundancy and fragile nodes, if any. You may confirm your conclusions by computational knock-off experiments.

This methodology seems particularly adapted to study larger networks with no or very little kinetic information. However, beyond that we have demonstrated that also ODE models may benefit from our complementary structural analysis approach because it may help to identify the core network, and thus contributes to model reduction. Moreover, model-based experiment design, specifically to explore a network's robustness, may benefit from the profound knowledge of a network's structure.

Our structuring method works for any type of network, independently of its functionality and its representation as a qualitative, time, stochastic, continuous, or hybrid Petri net. However, the reduction approach is based on steady-state oriented reasoning, and thus can only be applied in such circumstances.

Notably, the whole structural analysis approach is formally defined and supported by computational tools; no user interaction is required. So it works for networks of arbitrary size, and – because it requires only structural reasoning – also for (qualitatively) infinite state spaces. ADT sets can be directly computed, without having to compute the set of minimal T-invariants first. Thus the amount of T-invariants, which grows exponentially with the net size in the worst-case, does not establish a limiting factor.

## Methods

To be self-contained, we recall the basic Petri net notions relevant for this paper; for more details and formal definitions see [10], [20] and the supplementary material S1, for a general introduction into Petri net theory see [2]–[4].

### Biochemically Interpreted Petri Nets

**Figure 11:**
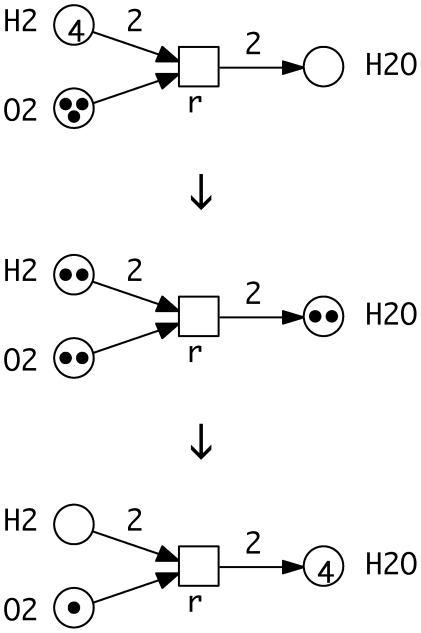
Petri net example. The Petri net for the well known chemical reaction r: 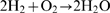 and three of its markings (states), connected each by a firing of the transition r. The transition is not enabled anymore in the marking reached after these two single firing steps.

To allow formal reasoning we represent biochemical networks by Petri nets, which combine executability and formal semantics amenable to mathematically sound analysis techniques. The idea to use Petri nets for the representation of biochemical networks is rather intuitive and has been mentioned by Carl Adam Petri himself in one of his internal research reports on interpretation of net theory in the seventies. It has also been used as the very first introductory example in one of the early survey papers [2]. We follow this approach, see Figure 11.

The standard notion of qualitative Petri nets are weighted, directed, bipartite graphs with the following basic ingredients.

**Figure 12:**
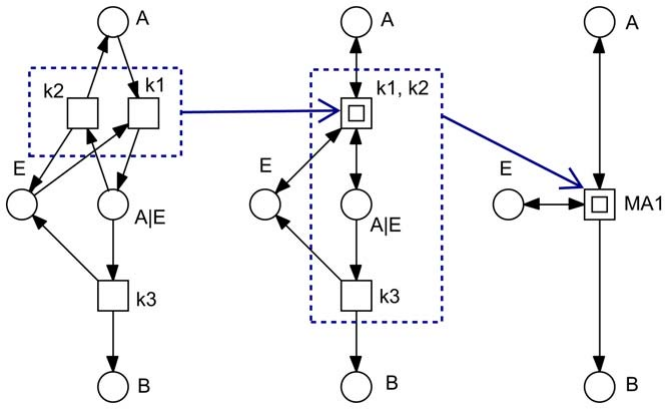
Hierarchical structuring by use of macro transitions. The three nets are identical and model a single enzymatic reaction following the mass-action kinetics 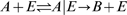. The transitions 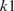 and 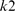 model a reversible reaction. Macro transitions (drawn as two centric squares) hide net details (in this case transition-bordered subnetworks) on the next lower hierarchy level. Macro transitions can be arbitrarily nested resulting into hierarchically structured Petri nets.

- There are two sets of nodes of distinguished type. The elements of one set are called *places* 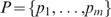, in the figures represented by circles, and the elements of the other set are called *transitions* 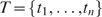, in the figures represented by rectangles. Places usually model passive system components like species playing the role of precursors and products of chemical reactions, while transitions stand for active system components like chemical reactions, transforming precursors into products. Reversible chemical reactions are modeled explicitly by two opposite transitions, compare Figure 12.
- The directed *arcs* connect always nodes of different type. They go from precursors to reactions, and from reactions to products. In other words, the pre-places of a transition correspond to the reaction's precursors, and its post-places to the reaction's products.
- Arcs are weighted by natural numbers. The arc weight may be read as the *multiplicity* of the arc, reflecting known stoichiometries, if any. The arc weight 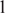 is the default value and is usually not given explicitly.
- A place carries an arbitrary number of *tokens*, represented as black dots or a natural number. The number zero is the default value and usually not given explicitly. Tokens can be interpreted as the available amount of a given species in number of molecules or moles, or any abstract, i.e. discrete concentration level. The tokens on all places establish the *marking* of the net, which represents the current state of the system.

The tokens may move through the net driven by the firing of transitions. The rules of the token game are defined by the firing rule. It consists of two parts: the precondition and the firing itself.

- A transition is *enabled*, if all its pre-places carry at least as many tokens as required by the weights of the corresponding ingoing arcs.
- An enabled transition *may* fire, i.e. a transition is never forced to fire. The firing of a transition removes from all its pre-places as many tokens as specified by the ingoing arc weights, and adds to all its post-places as many tokens as specified by the outgoing arc weights. The firing happens atomically and does not consume any time.

The repeated atomic firing of transitions establishes the discrete behavior of the qualitative Petri net, compare Figure 11. All markings, which can be reached from a given marking by any firing sequence of arbitrary length, constitute the *set of reachable markings*. The set of markings reachable from the initial marking is said to be the *state space* of a given Petri net. However, in this paper we confine ourselves deliberately to analysis techniques, which do not require the construction of the state space. So the presented approach works also for nets with infinite state spaces, i.e. for unbounded Petri nets.

We adopt the following drawing conventions, compare Figure 12.

- *Logical nodes* (fusion nodes) are colored in grey. All logical nodes with the same name are identical from an analysis point of view. They are commonly used for compounds involved in several reactions, in our case study for 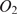.
- Transition-bordered subnets can be hidden in *macro transitions*, drawn as two centric squares. This allows an hierarchical structuring of larger nets. We apply this technique to coarsen a given net according to its minimal T-invariants' inherent structure.
- *Read arcs* (test arcs) are drawn as two opposite arcs. They are used to connect side conditions, which must be fulfilled for a reaction to take place, but which will not be changed by the firing. This applies in our case study to 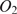.

### Invariant Analysis

#### Basic notions

The structure of a Petri net can be represented as a matrix, called incidence matrix in the Petri net community, and stoichiometric matrix in systems biology. The matrix representation opens the door to analysis techniques based on linear algebra (to be precise – discrete computational geometry). We recall the basic notions.

- The *incidence matrix* 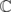 of a Petri net is an integer matrix with as many raws as there are places and as many columns as there are transitions. Raws and columns are indexed by 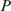 or 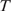, respectively. The matrix entry 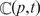 describes the token change on place 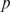 by the firing of transition 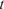.
- A *place vector* is a vector which has as many components as there are places; it is indexed by 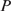. Likewise, a *transition vector* is a vector which has as many components as there are transitions; it is indexed by *T*.
- A *P-invariant* is a non-zero and non-negative integer place vector 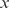 such that 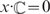; in words, for each transition it holds that: multiplying the P-invariant with the transition's column vector yields zero. Thus, the total effect of each transition on the P-invariant is zero, which explains its interpretation as a token-conserving component.
- A *T* *-invariant* is a non-zero and non-negative integer transition vector 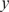 such that 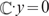; in words, for each place it holds that: multiplying the place's row with the T-invariant yields zero. Thus, the total effect of the T-invariant on a marking is zero, which explains its interpretation as a state-conserving component.
- The set of nodes corresponding to an invariant's non-zero entries are called the *support* of this invariant 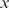, written as 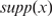.
- An invariant 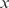 is called *minimal* if its support does not properly contain the support of another invariant, i.e. there is no invariant 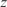 with 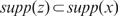, and the greatest common divisor of all non-zero entries of 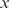 is 1.
- A net is *covered by P-invariants*, *shortly CPI*, (*covered by T* *-invariants*, *shortly CT* *I*) if every place (transition) belongs to a P-invariant (T-invariant).
- A *trivial T* *-invariant* consists of the two transitions modeling the two directions of a reversible reaction. A net which is covered by non-trivial T-invariants is said to be *strongly covered by T* *-invariants* (SCTI). Transitions not covered by non-trivial T-invariants are candidates for model reduction, e.g. if the model analysis is concerned with steady state analysis only.

The set 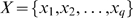 of all minimal P-invariants (T-invariants) of a given net is unique and represents a generating system for all P-invariants (T-invariants). All invariants 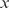 can be computed as non-negative linear combinations: 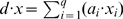, with 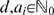, i.e. the allowed operations are addition, multiplication by a natural number, and division by a common divisor. Usually, we consider minimal invariants only.

A minimal P-invariant (T-invariant) defines a connected subnet, consisting of its support, its pre- and post-transitions (pre- and post-places), and all arcs in between. There are no structural limitations for such subnets induced by minimal invariants (for examples see [10]), but they are always connected, however not necessarily strongly connected.

Minimal invariants generally overlap; the combinatorial effect causes an explosion of the number of minimal invariants. There are exponentially many of them in the worst-case. Therefore we apply a structured representation of a given set of invariants, which is explained in the Section Structuring Method.

#### Applications

Invariants are a beneficial technique in model validation, and the challenge is to check all invariants for their biological plausibility. The minimal self-contained subnets induced by P-invariants or T-invariants, identify token-conserving or state-conserving modules, respectively, which should have an enclosed biological meaning.

A P-invariant stands for a set of places over which the weighted sum of tokens is constant and independent of any firing, i.e. for any markings 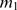, 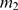, which are reachable by the firing of transitions, it holds that 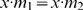. In the context of metabolic networks, P-invariants reflect substrate conservations, while in signal transduction or gene regulatory networks P-invariants often correspond to the several states of a given species (protein or protein complex) or gene. A place belonging to a P-invariant is obviously bounded, i.e. the number of tokens on each place is finite in any reachable marking, and CPI causes structural boundedness, i.e. boundedness for any initial marking.

The automatic identification of minimal T-invariants is in general useful as a method to highlight important parts of a network, and hence aid its comprehension by biochemists. Often, we are especially interested in a network's input/output behavior, which we characterize by input/output T-invariants (*I/O T* *-invariants*), i.e. such T-invariants, involving input and output transitions, or places considered to be input/output species. These special T-invariants can often be read as alternative, self-contained pathways within a given network.

#### Structural deadlock

A notion related to P-invariants is *structural deadlock*. A non-empty set of places 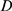 is called structural deadlock if the set of pre-transitions of 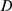 is contained in the set of post-transitions of 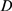, i.e. every transition, which fires tokens onto a place in this structural deadlock set 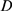, also has a pre-place in 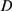.

Pre-transitions of a structural deadlock cannot fire if the structural deadlock is clean, i.e. it does not contain a token. Therefore, a structural deadlock can not get tokens again as soon as it got clean, and then all its post-transitions are dead. Consequently, a structural deadlock needs a non-empty initial marking.

The support of a P-invariant defines a structural deadlock, but not vice versa.

### Structuring Method

The following discussion concentrates on T-invariants. Likewise, the technique can be applied to P-invariants due to the given symmetry of the two notions.

We introduce a *dependency relation* based on a set of minimal T-invariants. It can be equally applied to the full set of all minimal T-invariants as well as to a subset, e.g. the set of non-trivial T-invariants. Let 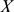 denote a set of minimal T-invariants 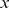 of a given Petri net. Two transitions 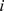, 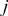 depend on each other, if
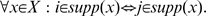



This is an abstract dependency defined on the invariants' support. Dependent transitions appear always together in the given set of minimal T-invariants. The knock out of one of them prevents the whole set of transitions depending on each other to accomplish their common function. The dependency relation is reflexive, symmetric, and transitive. Thus it is an equivalence relation in the transition set 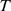, leading to a partition of 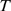. We call the equivalence classes 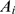 maximal *abstract dependent transition sets* (ADT sets). The classification of all transitions is based on the T-invariants' supports, and it holds
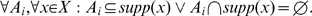



Contrary to T-invariants, which generally overlap, ADT sets are disjunctive by definition and induce subnets which may overlap in interface places only.

The subnets induced by ADT sets represent a possible structural decomposition of biochemical networks into smaller subnets, which can be read as the smallest biologically meaningful functional units or building blocks a network is composed of. The decomposition is formally defined, requires no user interaction, and is only based on statically decidable properties. We hide building blocks defined by ADT sets within macro transitions which helps to hierarchical structure larger networks. However, maximal ADT sets are not necessarily connected. Hence, a further decomposition into connected ADT sets is generally needed; then we get non-maximal ADT sets. Having a decomposition of the set of transitions into ADT sets inducing connected subnets, we are able to coarsen automatically a given net according to the minimal T-invariants' inherent structure:

- macro transitions abstract from connected ADT sets, and
- places on the hierarchy's top level correspond to the interface between the ADT sets.

The coarse structure gives a structured representation of all T-invariants, which may contribute to a better understanding of the net behavior. Moreover, the coarse structure allows to identify sensitive net parts, i.e. the knock out of which would switch off a significant part of the network, a whole pathway or even prevent any output; see [20] for a more detailed discussion.

Notably, ADT sets can be directly computed, without having to compute the set of minimal T-invariants first. This can be done by checking the following system of linear equations for solvability for all pairs of transitions 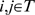:
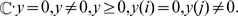
Thus the amount of T-invariants, which grows exponentially with the net size in the worst-case, does not establish a limiting factor for our hierarchical structuring approach.

### Continuous Petri Nets

This work involves the differential equations formalism. For this purpose we read the constructed qualitative Petri nets describing the hypoxia network as continuous Petri nets [10], [30].

In a continuous Petri net the marking of a place is no longer an integer, but a positive real number. It is called token value, which we interpret as the concentration of the species modeled by the place. Due to the influence of time, a continuous transition is forced to fire as soon as possible. The instantaneous firing of a transition is carried out like a continuous flow, whereby the strength of the flow is determined by the transition's *firing rate function*. The current deterministic firing rate generally depends on the current marking of the pre-places, i.e. of the current concentrations of the reaction's precursors. In the case of mass-action kinetics, a transition's rate function is given by the product of its pre-places (read as real-valued variables) and its kinetic constant.

A continuous Petri net uniquely defines a system of ordinary differential equations (ODEs), where one equation describes the continuous change over time on the token value of a given place by the continuous increase of its pre-transitions' flow and the continuous decrease of its post-transitions' flow, i.e., each place 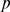 subject to changes (i.e. dynamic variables) generates an equation:
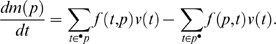



Please note, the notation 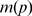 refers to the current token value of place 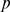 and corresponds to the more popular notation 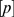. 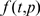 specifies the weight of the arc going from transition 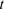 to place 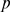, 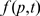 the weight of the arc going from place 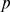 to transition 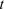, and 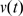 the rate function of 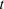. 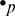 yields the set of pre-transitions of 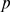, and 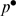 yields the set of post-transitions of 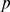.

Each equation corresponds basically to a line in the incidence matrix, whereby now the matrix elements consist of the rate functions multiplied by the arc weight, if any. Moreover, as soon as there are transitions with more than one pre-place, we get typically a non-linear system, which calls for a simulative solution by numerical integration of the system on hand. With other words, the continuous Petri net becomes the structured description of the corresponding ODEs, see also [10], [11], [30]. The correspondence between the graphical notations, Petri nets and the differential equations terminologies is summarized in Table 6.

**Table 6:** Correspondence between Petri nets and ODEs.

Graphics	Petri nets	Bio semantics	ODEs
circles	places	species	variables
squares	transitions	reactions	rates

### Supporting Information

The Petri net tools used in this case study as well as all Petri net versions of the hypoxia response network are available at http://www-dssz.informatik.tu-cottbus.de.

We also provide self-contained supplementary material (see S1 on the journal website) with the formal definitions of all technical terms of the Petri net theory used in this paper.

## Supporting Information

Supplementary Material S1 Formal definitions for the Method section.
(0.08 MB PDF)
