# Supplementary material for: Structural Analysis to Determine the Core of Hypoxia Response Network
Source: PLoS One. 2010 Jan 19;5(1):e8600. doi: 10.1371/journal.pone.0008600 (PMC2808224; doi:10.1371/journal.pone.0008600)
Supplement: Supplementary Material S1 — Formal definitions for the Method section. (0.08 MB PDF) [file pone.0008600.s001.pdf]

Supplementary material

Monika Heiner, K. Sriram:
Structural Analysis to Determine the Core of Hypoxia Response Network,
PLoS ONE, December 2009

Methods

In the following we recall the basic Petri net notions and give the formal definitions relevant for this paper; for more details see [1, 2], for a general introduction into Petri net theory see [3, 4], for theoretical issues see the excellent textbook [5], which however is in German.

As usual, we denote the set of non-negative integers including zero by $\mathbb{N}_0$, the set of integers by $\mathbb{Z}$, and the set of non-negative real numbers by $\mathbb{R}_0^+$. $|S|$ denotes the number of elements in a set S.

Biochemically Interpreted Petri Nets

To allow formal reasoning we represent biochemical networks by Petri nets, which combine executability and formal semantics amenable to mathematically sound analysis techniques.

Definition 1 (Petri net, Syntax). A Petri net is a quadruple $\mathcal{N} = (P, T, f, m_0)$, where

- P and T are finite sets with $P \cup T \neq \emptyset$, $P \cap T = \emptyset$,
- $f : ((P \times T) \cup (T \times P)) \to \mathbb{N}_0$,
- $m_0 : P \to \mathbb{N}_0$. □

The elements of P are called *places* and the elements of T *transitions*. The function f specifies the arcs and their non-negative integer weights, m_0 the *initial marking* (state). $m(p)$ yields the number of tokens on place p in the marking m. We introduce the following notions and notations for a node $x \in P \cup T$.

- ${}^\bullet x := \{y \in P \cup T | f(y, x) \neq 0\}$ is the pre-set of x.
- $x^\bullet := \{y \in P \cup T | f(x, y) \neq 0\}$ is the post-set of x.
- x is called input node if ${}^\bullet x = \emptyset$.
- x is called output node if $x^\bullet = \emptyset$.
- x is called boundary node if it is either input or output node.

We extend the first two notions to a set of nodes $X \subseteq P \cup T$ and define:

- the set of all pre-nodes by ${}^\bullet X := \bigcup_{x \in X} {}^\bullet x$, and
- the set of all post-nodes by $X^\bullet := \bigcup_{x \in X} x^\bullet$.

Up to now we have specified the static aspects of a Petri net. The behavior of a net is defined by the firing rule, which consists of two parts: the precondition and the firing itself.

Definition 2 (Petri net, Firing rule). Let $\mathcal{N} = (P, T, f, m_0)$ be a Petri net.

- A transition t is enabled in a marking m, written as $m[t\rangle$, if
  $\forall p \in {}^\bullet t : m(p) \geq f(p, t)$, else disabled.

- A transition t, which is enabled in m, may fire.
- When t in m fires, a new marking m' is reached, written as $m[t\rangle m'$, with $\forall p \in P : m'(p) = m(p) - f(p,t) + f(t,p)$.
- The firing happens atomically and does not consume time. □

The repeated firing of transitions establishes the behavior of the net. The whole net behavior consists of all possible partially ordered firing sequences (partial order semantics), or all possible totally ordered firing sequences (interleaving semantics), respectively.

Every marking m is defined by the given token situation in all places, i.e. $m \in \mathbb{N}_0^{|P|}$. All markings, which can be reached from a given marking m by any firing sequence of arbitrary length, constitute the *set of reachable markings* $[m\rangle$. The set of markings $[m_0\rangle$ reachable from the initial marking is said to be the *state space* of a given Petri net.

Invariant Analysis

Basic notions

The structure of a Petri net can be represented as a matrix, called incidence matrix in the Petri net community, and stoichiometric matrix in systems biology. The matrix representation opens the door to analysis techniques based on linear algebra (to be precise – discrete computational geometry). We recall the basic notions.

Definition 3 (P-invariants, T-invariants). Let $\mathcal{N} = (P, T, f, m_0)$ be a Petri net.

- The incidence matrix of $\mathcal{N}$ is a matrix $\mathbb{C} : P \times T \to \mathbb{Z}$, indexed by P and T, such that $\mathbb{C}(p,t) = f(t,p) - f(p,t)$.
- A place vector (transition vector) is a vector $x : P \to \mathbb{Z}$, indexed by P ($y : T \to \mathbb{Z}$, indexed by T).
- A place vector (transition vector) is called a P-invariant (T-invariant) if it is a non-trivial non-negative integer solution of the homogenous linear equation system $x \cdot \mathbb{C} = 0$ ($\mathbb{C} \cdot y = 0$).
- The set of nodes corresponding to an invariant's non-zero entries are called the support of this invariant x, written as $supp\,(x)$.
- An invariant x is called minimal if there is no invariant z with $supp\,(z) \subset supp\,(x)$, and the greatest common divisor of all non-zero entries of x is 1.
- A net is covered by P-invariants, shortly CPI, (covered by T-invariants, shortly CTI) if every place (transition) belongs to a P-invariant (T-invariant). □

Invariants are vectors, which can be read as specifications of multi-sets. Contrary, supports are sets, which can technically be specified as vectors over Booleans, which allows the access to the ith entry by indexing.

The set $X = \{x_1, x_2, \ldots, x_q\}$ of all minimal P-invariants (T-invariants) of a given net is unique and represents a generating system for all P-invariants (T-invariants). All invariants x can be computed as non-negative linear combinations:

$$d \cdot x = \sum_{i=1}^{q} (a_i \cdot x_i), \;\; d, a_i \in \mathbb{N}_0 \,. \qquad (1)$$

We conclude with two observations.

- A minimal P-invariant (T-invariant) defines a connected subnet, consisting of its support, its pre- and post-transitions (pre- and post-places), and all arcs in between. There are no structural limitations for such subnets induced by minimal invariants (for examples see [1]), but they are always connected, however not necessarily strongly connected.
- Minimal invariants generally overlap; the combinatorial effect causes an explosion of the number of minimal invariants. There are exponentially many of them in the worst-case. Therefore we apply a structured representation of a given set of invariants, which is explained in the Section Structuring Method.

Applications

The minimal self-contained subnets induced by P-invariants or T-invariants, identify token-conserving or state-conserving modules, respectively, which should have an enclosed (biological) meaning.

A *P-invariant* x stands for a set of places over which the weighted sum of tokens is constant and independent of any firing, i.e. for any markings m_1, m_2, which are reachable by the firing of transitions, it holds that $x \cdot m_1 = x \cdot m_2$. A place belonging to a P-invariant is obviously bounded, i.e. the number of tokens on each place is finite in any reachable marking. Thus, CPI causes structural boundedness, i.e. boundedness for any initial marking.

A *T-invariant* y has two interpretations in the context of biochemical networks.

- The entries of a T-invariant specify a multi-set of transitions which by their partially ordered firing reproduce a given marking, i.e. basically occurring one after the other. This partial order sequence of the T-invariant's transitions may contribute to a deeper understanding of the net behavior. A T-invariant is called *feasible* if such a behavior is actually possible in the given marking situation.
- The entries of a T-invariant may also be read as the relative firing rates of transitions, all of them occurring permanently and concurrently. This activity level (rate) corresponds to the steady state behavior.

The two transitions modeling the two directions of a reversible reaction always make a minimal T-invariant; thus it is called *trivial T-invariant.* A net which is covered by non-trivial T-invariants is said to be *strongly covered by T-invariants* (SCTI). Transitions not covered by non-trivial T-invariants are candidates for model reduction, e.g. if the model analysis is concerned with steady state analysis only.

Structural deadlock

A notion related to P-invariants is *structural deadlock.*

Definition 4 (Structural deadlock). Let $\mathcal{N} = (P, T, f, m_0)$ be a Petri net.

- A non-empty set of places $D \subseteq P$ is called *structural deadlock* if ${}^\bullet D \subseteq D^\bullet$. □

Every transition which fires tokens onto a place in the structural deadlock set D, also has a pre-place in this set D. Thus, pre-transitions of a structural deadlock cannot fire if the structural deadlock is clean, i.e. does not contain a token. Therefore, a structural deadlock cannot get tokens again as soon as it got clean, and then all its post-transitions $t \in D^\bullet$ are dead. Consequently, a structural deadlock needs a non-empty initial marking.

For a P-invariant x it holds ${}^\bullet supp(x) = supp(x)^\bullet$. Thus, the support of a P-invariant is always a structural deadlock, but not vice versa.

Structuring Method

The following discussion concentrates on T-invariants. Likewise, the technique can be applied to P-invariants due to the given symmetry of the two notions.

We define a dependency relation based on a set of minimal T-invariants. It can be equally applied to the full set of all minimal T-invariants as well as to a subset, e.g. the set of non-trivial T-invariants.

Definition 5 (Dependency relation). Let $\mathcal{N} = (P, T, f, m_0)$ be a Petri net, and let X denote a set of minimal T-invariants x of $\mathcal{N}$. Two transitions i, $j \in T$ depend on each other, $i \bowtie j$ for short, if

$$\forall x \in X : i \in supp(x) \Leftrightarrow j \in supp(x). \qquad \square$$

The dependency relation fulfills the following properties:

- reflexivity: $i \bowtie i$;
  a transition depends on its own.
- symmetry: $i \bowtie j \Leftrightarrow j \bowtie i$;
  the dependency of i on j implies the dependency of j on i, and vice versa.
- transitivity: $i \bowtie j \wedge j \bowtie k \Rightarrow i \bowtie k$;
  if i depends on j, and j depends on k, then i depends also on k.

Thus it is an equivalence relation in the transition set T, leading to a partition of T. We call the equivalence classes A_i with

$$A_i \subseteq T \ \wedge \ \cup A_i = T \ \wedge \ \forall i, j : i \neq j \Rightarrow A_i \cap A_j = \emptyset \, , \tag{2}$$

maximal *abstract dependent transition sets* (ADT sets). The classification of all transitions is based on the T-invariants' supports only, and it holds

$$\forall A_i, \forall x \in X : A_i \subseteq supp(x) \ \vee \ A_i \cap supp(x) = \emptyset. \tag{3}$$

Contrary to T-invariants, which generally overlap, ADT sets are disjunctive by definition and induce subnets which may overlap in interface places only. The set of interface places P_{IF} between two ADT sets A_i and A_j, with $i \neq j$, is formally defined by

$$P_{IF} = (^\bullet A_i \cup A_i{}^\bullet) \cap (^\bullet A_j \cup A_j{}^\bullet) \subseteq P \, . \tag{4}$$

The subnets induced by ADT sets represent a possible structural decomposition of networks into smaller subnets, which however are not necessarily connected. Generally, a further decomposition into connected ADT sets is needed. Then we get non-maximal ADT sets. The decomposition of the set of transitions into ADT sets inducing connected subnets guides the coarsening of a given net:

- macro transitions abstract from connected ADT sets, and
- places on the hierarchy's top level correspond to the interface between the ADT sets.

The coarse structure gives a structured representation of all T-invariants, which may contribute to a better understanding of the net behavior, see [2] for a more detailed discussion.

Notably, ADT sets can be directly computed, without having to compute the set of minimal T-invariants first. This can be done by checking the following system of linear equations for solvability for all pairs of transitions $i, j \in T$:

$$\mathbb{C} \cdot y = 0, y \neq 0, y \geq 0, y(i) = 0, y(j) \neq 0\,. \tag{5}$$

Thus the amount of T-invariants, which grows exponentially with the net size in the worst-case, does not establish a limiting factor for our hierarchical structuring approach.

Continuous Petri Nets

Continuous Petri net, Introduction

Continuous Petri nets [6,7] are a quantified version of the standard notion of qualitative Petri nets. Like their ancestor, they are weighted, directed, bipartite graphs, however arc weights and the numbers assigned to places are now non-negative real numbers.

Definition 6 (Continuous Petri net, Syntax). A biochemically interpreted continuous Petri net is a quintuple $\mathcal{CON}_{Bio} = (P, T, f, m_0, v)$, where

- P and T are finite sets with $P \cup T \neq \emptyset$, $P \cap T = \emptyset$,
- $f : ((P \times T) \cup (T \times P)) \to R_0^+$,
- $m_0 : P \to R_0^+$.
- $v : T \to H$ with
  - $H := \{h_t \,|\, h_t : R^{|^\bullet t|} \to R\,,\, t \in T\}$,
  - $v(t) = h_t$ for all transitions $t \in T$. □

The elements of P are called *continuous places* and the elements of T *continuous transitions*. The function f specifies the arcs and their non-negative real-valued weights, m_0 the *initial continuous marking* (state). $m(p) \in \mathbb{R}_0^+$ yields the token value, which we interpret as the concentration of the species modeled by the place. Please note, $m(p)$ corresponds to $[\,p\,]$, a notation more popular in systems biology.

H is the set of all *firing rate functions* h_t, and the function v assigns to each transition t a firing rate function h_t. The domain of h_t is restricted to the set of pre-places of t to enforce a close relation between network structure and firing rate functions. Therefore, the marking-dependent continuous firing rate $h_t(m)$ actually depends only on a sub-marking.

Technically, any mathematical function in compliance with this restriction is allowed for h_t. However, often special kinetic patterns are applied, whereby Michaelis-Menten and mass-action kinetics seem to be the most popular ones.

A firing rate may also be negative, in which case the reaction takes place in the reverse direction. This feature is commonly used to model reversible reactions by just one transition, where (per definition) positive firing rates correspond to the forward direction, and negative ones to the backward direction.

We adopt for continuous nodes all notions and notations, which have been introduced for discrete nodes (right after Definition 1).

Continuous Petri net, Semantics

Each continuous marking is a place vector $m \in (\mathbb{R}_0^+)^{|P|}$. A continuous transition t is enabled in m, if $\forall p \in {}^\bullet t : m(p) > 0$. Due to the influence of time, a continuous transition is forced to fire as soon as possible. The instantaneous firing of a transition t is carried out like a continuous flow, whereby the strength of the flow is determined by its firing rate function $v(t)$.

The semantics of a continuous Petri net is defined by a system of ODEs, whereby one equation describes the continuous change over time on the token value of a given place by the continuous increase of its pre-transitions' flow and the continuous decrease of its post-transitions' flow, i.e., each place p subject to changes gets its own equation:

$$\frac{dm\,(p)}{dt} = \sum_{t \in {}^\bullet p} f\,(t,p)\,v\,(t) - \sum_{t \in p^\bullet} f\,(p,t)\,v\,(t)\ . \tag{6}$$

Each equation corresponds basically to a line in the incidence matrix, whereby now the matrix elements consist of the rate functions multiplied by the arc weights, if any.

In other words, the continuous Petri net becomes the structured description of the corresponding ODEs, see also [1, 7, 8]. The ODEs defined by a continuous Petri net are unique (up to behavior-preserving mathematical transformations), but not vice versa. Generally, the network structure is not uniquely defined by a system of ODEs.

References

1. Heiner M, Gilbert D, Donaldson R (2008) Petri nets in systems and synthetic biology. In: Schools on Formal Methods (SFM). LNCS 5016, Springer, pp. 215-264.

2. Heiner M (2009) Understanding network behaviour by structured representations of transition invariants. In: Algorithmic Bioprocesses. Springer, Natural Computing series, pp. 367–389.

3. Murata T (1989) Petri nets: Properties, analysis and applications. Proceedings of the IEEE 77: 541–580.

4. Bause F, Kritzinger P (2002) Stochastic Petri Nets. Vieweg.

5. Priese L, Wimmel H (2003) Theoretical Informatics - Petri Nets (in German). Springer.

6. David R, Alla H (2005) Discrete, Continuous, and Hybrid Petri Nets. Springer.

7. Gilbert D, Heiner M (2006) From Petri nets to differential equations - an integrative approach for biochemical network analysis. In: Proc. ICATPN 2006. LNCS 4024, Springer, pp. 181–200.

8. Breitling R, Gilbert D, Heiner M, Orton R (2008) A structured approach for the engineering of biochemical network models, illustrated for signalling pathways. Briefings in Bioinformatics : 404 – 421.
